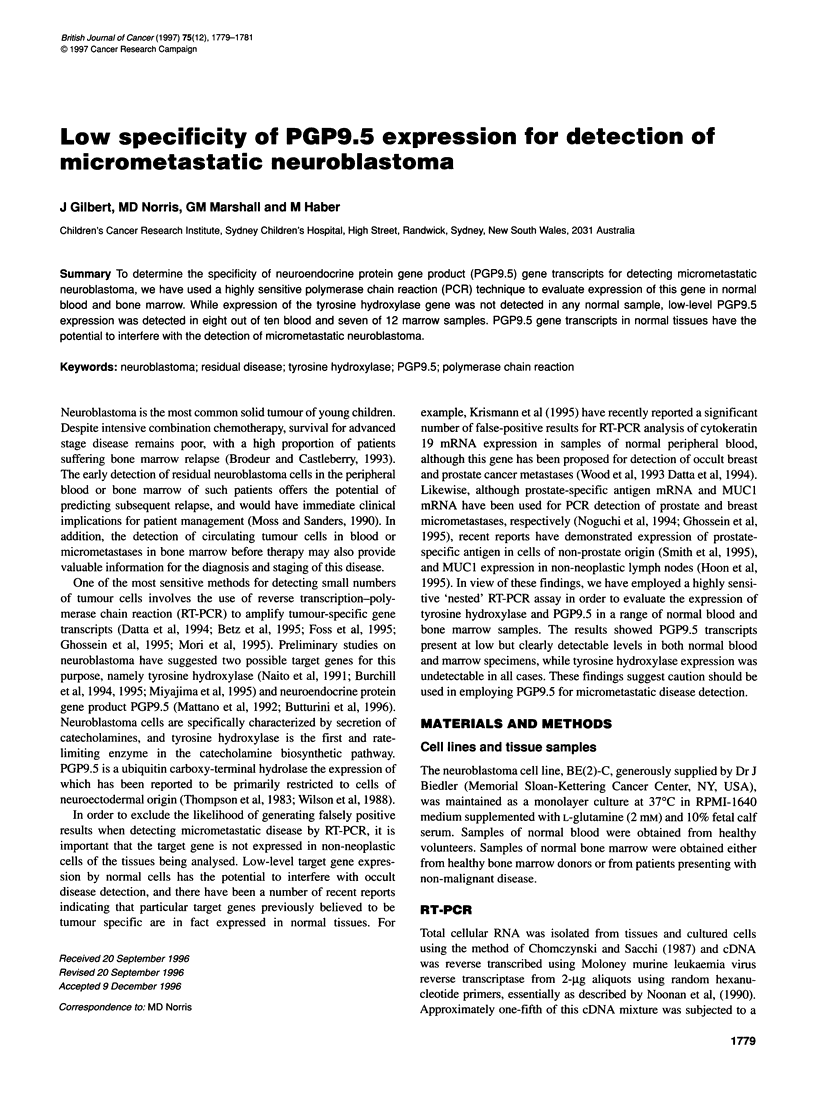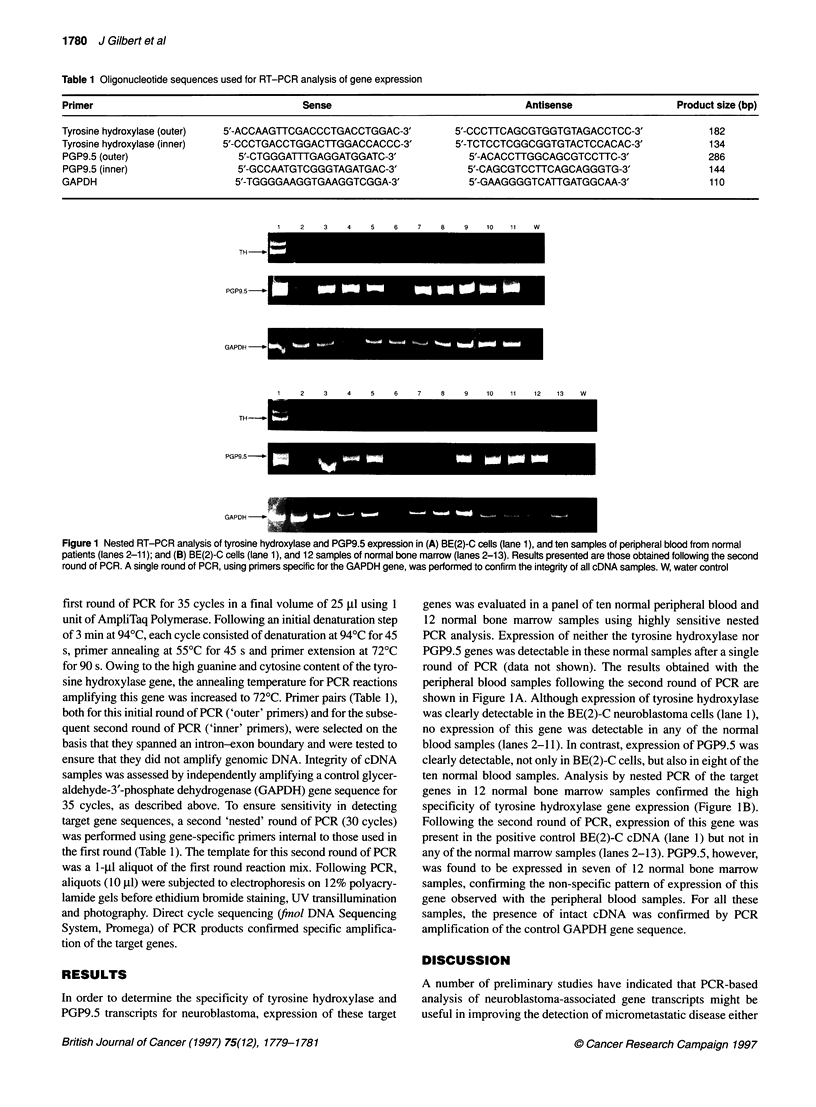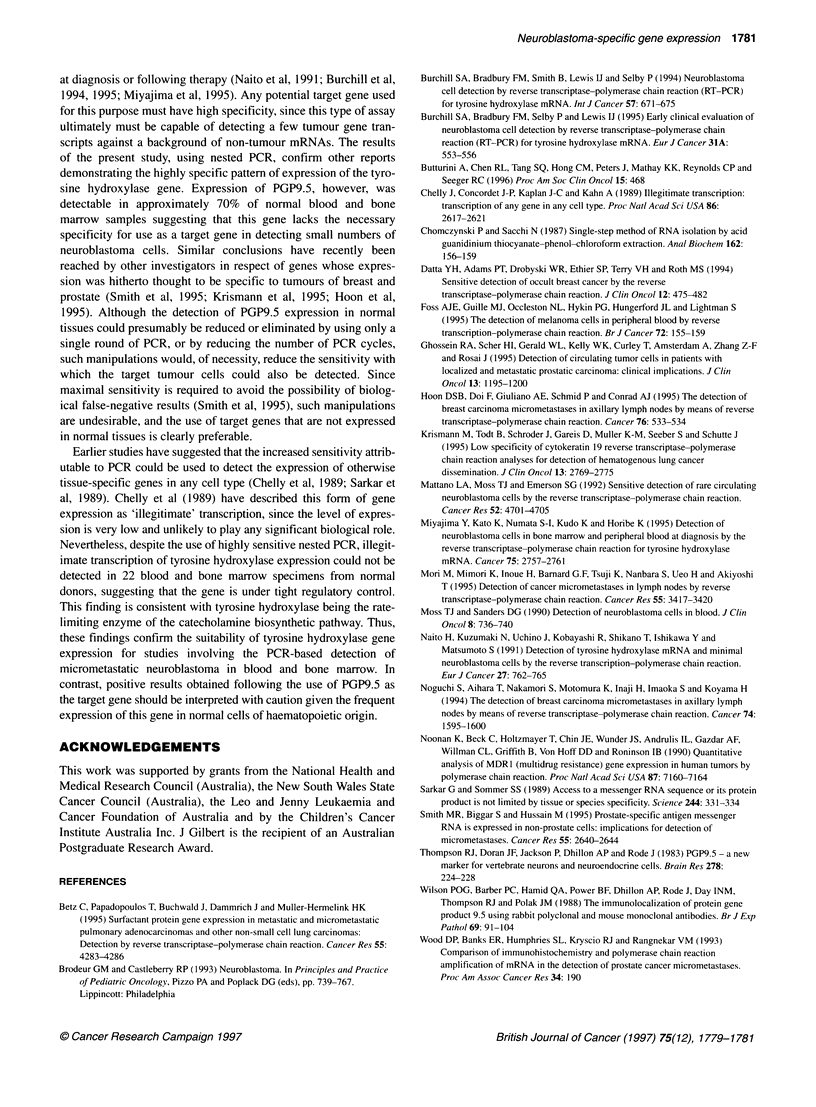# Low specificity of PGP9.5 expression for detection of micrometastatic neuroblastoma.

**DOI:** 10.1038/bjc.1997.303

**Published:** 1997

**Authors:** J. Gilbert, M. D. Norris, G. M. Marshall, M. Haber

**Affiliations:** Children's Cancer Research Institute, Sydney Children's Hospital, New South Wales, Australia.

## Abstract

**Images:**


British Journal of Cancer (1997) 75(12), 1779–1781
© 1997 Cancer Research Campaign

### Low specificity of PGP9.5 expression for detection of micrometastatic neuroblastoma

**J Gilbert, MD Norris, GM Marshall and M Haber**

Children's Cancer Research Institute, Sydney Children's Hospital, High Street, Randwick, Sydney, New South Wales, 2031 Australia

**Summary** To determine the specificity of neuroendocrine protein gene product (PGP9.5) gene transcripts for detecting micrometastatic neuroblastoma, we have used a highly sensitive polymerase chain reaction (PCR) technique to evaluate expression of this gene in normal blood and bone marrow. While expression of the tyrosine hydroxylase gene was not detected in any normal sample, low-level PGP9.5 expression was detected in eight out of ten blood and seven of 12 marrow samples. PGP9.5 gene transcripts in normal tissues have the potential to interfere with the detection of micrometastatic neuroblastoma.

**Keywords:** neuroblastoma; residual disease; tyrosine hydroxylase; PGP9.5; polymerase chain reaction

Neuroblastoma is the most common solid tumour of young children. Despite intensive combination chemotherapy, survival for advanced stage disease remains poor, with a high proportion of patients suffering bone marrow relapse (Brodeur and Castleberry, 1993). The early detection of residual neuroblastoma cells in the peripheral blood or bone marrow of such patients offers the potential of predicting subsequent relapse, and would have immediate clinical implications for patient management (Moss and Sanders, 1990). In addition, the detection of circulating tumour cells in blood or micrometastases in bone marrow before therapy may also provide valuable information for the diagnosis and staging of this disease.

One of the most sensitive methods for detecting small numbers of tumour cells involves the use of reverse transcription–polymerase chain reaction (RT-PCR) to amplify tumour-specific gene transcripts (Datta et al, 1994; Betz et al, 1995; Foss et al, 1995; Ghossein et al, 1995; Mori et al, 1995). Preliminary studies on neuroblastoma have suggested two possible target genes for this purpose, namely tyrosine hydroxylase (Naito et al, 1991; Burchill et al, 1994, 1995; Miyajima et al, 1995) and neuroendocrine protein gene product PGP9.5 (Mattano et al, 1992; Butturini et al, 1996). Neuroblastoma cells are specifically characterized by secretion of catecholamines, and tyrosine hydroxylase is the first and rate-limiting enzyme in the catecholamine biosynthetic pathway. PGP9.5 is a ubiquitin carboxy-terminal hydrolase the expression of which has been reported to be primarily restricted to cells of neuroectodermal origin (Thompson et al, 1983; Wilson et al, 1988).

In order to exclude the likelihood of generating falsely positive results when detecting micrometastatic disease by RT-PCR, it is important that the target gene is not expressed in non-neoplastic cells of the tissues being analysed. Low-level target gene expression by normal cells has the potential to interfere with occult disease detection, and there have been a number of recent reports indicating that particular target genes previously believed to be tumour specific are in fact expressed in normal tissues. For example, Krismann et al (1995) have recently reported a significant number of false-positive results for RT-PCR analysis of cytokeratin 19 mRNA expression in samples of normal peripheral blood, although this gene has been proposed for detection of occult breast and prostate cancer metastases (Wood et al, 1993 Datta et al, 1994). Likewise, although prostate-specific antigen mRNA and MUC1 mRNA have been used for PCR detection of prostate and breast micrometastases, respectively (Noguchi et al, 1994; Ghossein et al, 1995), recent reports have demonstrated expression of prostate-specific antigen in cells of non-prostate origin (Smith et al, 1995), and MUC1 expression in non-neoplastic lymph nodes (Hoon et al, 1995). In view of these findings, we have employed a highly sensitive 'nested' RT-PCR assay in order to evaluate the expression of tyrosine hydroxylase and PGP9.5 in a range of normal blood and bone marrow samples. The results showed PGP9.5 transcripts present at low but clearly detectable levels in both normal blood and marrow specimens, while tyrosine hydroxylase expression was undetectable in all cases. These findings suggest caution should be used in employing PGP9.5 for micrometastatic disease detection.

*Received 20 September 1996*
*Revised 20 September 1996*
*Accepted 9 December 1996*

*Correspondence to:* MD Norris

### MATERIALS AND METHODS

### Cell lines and tissue samples

The neuroblastoma cell line, BE(2)-C, generously supplied by Dr J Biedler (Memorial Sloan-Kettering Cancer Center, NY, USA), was maintained as a monolayer culture at 37°C in RPMI-1640 medium supplemented with L-glutamine (2 mM) and 10% fetal calf serum. Samples of normal blood were obtained from healthy volunteers. Samples of normal bone marrow were obtained either from healthy bone marrow donors or from patients presenting with non-malignant disease.

### RT-PCR

Total cellular RNA was isolated from tissues and cultured cells using the method of Chomczynski and Sacchi (1987) and cDNA was reverse transcribed using Moloney murine leukaemia virus reverse transcriptase from 2-μg aliquots using random hexanucleotide primers, essentially as described by Noonan et al, (1990). Approximately one-fifth of this cDNA mixture was subjected to a

**Table 1** Oligonucleotide sequences used for RT–PCR analysis of gene expression

| Primer | Sense | Antisense | Product size (bp) |
|---|---|---|---|
| Tyrosine hydroxylase (outer) | 5′-ACCAAGTTCGACCCTGACCTGGAC-3′ | 5′-CCCTTCAGCGTGGTGTAGACCTCC-3′ | 182 |
| Tyrosine hydroxylase (inner) | 5′-CCCTGACCTGGACTTGGACCACCC-3′ | 5′-TCTCCTCGGCGGTGTACTCCACAC-3′ | 134 |
| PGP9.5 (outer) | 5′-CTGGGATTTGAGGATGGATC-3′ | 5′-ACACCTTGGCAGCGTCCTTC-3′ | 286 |
| PGP9.5 (inner) | 5′-GCCAATGTCGGGTAGATGAC-3′ | 5′-CAGCGTCCTTCAGCAGGGTG-3′ | 144 |
| GAPDH | 5′-TGGGGAAGGTGAAGGTCGGA-3′ | 5′-GAAGGGGTCATTGATGGCAA-3′ | 110 |

**Figure 1** Nested RT–PCR analysis of tyrosine hydroxylase and PGP9.5 expression in (**A**) BE(2)-C cells (lane 1), and ten samples of peripheral blood from normal patients (lanes 2–11); and (**B**) BE(2)-C cells (lane 1), and 12 samples of normal bone marrow (lanes 2–13). Results presented are those obtained following the second round of PCR. A single round of PCR, using primers specific for the GAPDH gene, was performed to confirm the integrity of all cDNA samples. W, water control first round of PCR for 35 cycles in a final volume of 25 μl using 1 unit of AmpliTaq Polymerase. Following an initial denaturation step of 3 min at 94°C, each cycle consisted of denaturation at 94°C for 45 s, primer annealing at 55°C for 45 s and primer extension at 72°C for 90 s. Owing to the high guanine and cytosine content of the tyrosine hydroxylase gene, the annealing temperature for PCR reactions amplifying this gene was increased to 72°C. Primer pairs (Table 1), both for this initial round of PCR ('outer' primers) and for the subsequent second round of PCR ('inner' primers), were selected on the basis that they spanned an intron–exon boundary and were tested to ensure that they did not amplify genomic DNA. Integrity of cDNA samples was assessed by independently amplifying a control glyceraldehyde-3′-phosphate dehydrogenase (GAPDH) gene sequence for 35 cycles, as described above. To ensure sensitivity in detecting target gene sequences, a second 'nested' round of PCR (30 cycles) was performed using gene-specific primers internal to those used in the first round (Table 1). The template for this second round of PCR was a 1-μl aliquot of the first round reaction mix. Following PCR, aliquots (10 μl) were subjected to electrophoresis on 12% polyacrylamide gels before ethidium bromide staining, UV transillumination and photography. Direct cycle sequencing (*fmol* DNA Sequencing System, Promega) of PCR products confirmed specific amplification of the target genes.

### RESULTS

In order to determine the specificity of tyrosine hydroxylase and PGP9.5 transcripts for neuroblastoma, expression of these target genes was evaluated in a panel of ten normal peripheral blood and 12 normal bone marrow samples using highly sensitive nested PCR analysis. Expression of neither the tyrosine hydroxylase nor PGP9.5 genes was detectable in these normal samples after a single round of PCR (data not shown). The results obtained with the peripheral blood samples following the second round of PCR are shown in Figure 1A. Although expression of tyrosine hydroxylase was clearly detectable in the BE(2)-C neuroblastoma cells (lane 1), no expression of this gene was detectable in any of the normal blood samples (lanes 2–11). In contrast, expression of PGP9.5 was clearly detectable, not only in BE(2)-C cells, but also in eight of the ten normal blood samples. Analysis by nested PCR of the target genes in 12 normal bone marrow samples confirmed the high specificity of tyrosine hydroxylase gene expression (Figure 1B). Following the second round of PCR, expression of this gene was present in the positive control BE(2)-C cDNA (lane 1) but not in any of the normal marrow samples (lanes 2–13). PGP9.5, however, was found to be expressed in seven of 12 normal bone marrow samples, confirming the non-specific pattern of expression of this gene observed with the peripheral blood samples. For all these samples, the presence of intact cDNA was confirmed by PCR amplification of the control GAPDH gene sequence.

### DISCUSSION

A number of preliminary studies have indicated that PCR-based analysis of neuroblastoma-associated gene transcripts might be useful in improving the detection of micrometastatic disease either

 © Cancer Research Campaign 1997

at diagnosis or following therapy (Naito et al, 1991; Burchill et al, 1994, 1995; Miyajima et al, 1995). Any potential target gene used for this purpose must have high specificity, since this type of assay ultimately must be capable of detecting a few tumour gene transcripts against a background of non-tumour mRNAs. The results of the present study, using nested PCR, confirm other reports demonstrating the highly specific pattern of expression of the tyrosine hydroxylase gene. Expression of PGP9.5, however, was detectable in approximately 70% of normal blood and bone marrow samples suggesting that this gene lacks the necessary specificity for use as a target gene in detecting small numbers of neuroblastoma cells. Similar conclusions have recently been reached by other investigators in respect of genes whose expression was hitherto thought to be specific to tumours of breast and prostate (Smith et al, 1995; Krismann et al, 1995; Hoon et al, 1995). Although the detection of PGP9.5 expression in normal tissues could presumably be reduced or eliminated by using only a single round of PCR, or by reducing the number of PCR cycles, such manipulations would, of necessity, reduce the sensitivity with which the target tumour cells could also be detected. Since maximal sensitivity is required to avoid the possibility of biological false-negative results (Smith et al, 1995), such manipulations are undesirable, and the use of target genes that are not expressed in normal tissues is clearly preferable.

Earlier studies have suggested that the increased sensitivity attributable to PCR could be used to detect the expression of otherwise tissue-specific genes in any cell type (Chelly et al, 1989; Sarkar et al, 1989). Chelly et al (1989) have described this form of gene expression as 'illegitimate' transcription, since the level of expression is very low and unlikely to play any significant biological role. Nevertheless, despite the use of highly sensitive nested PCR, illegitimate transcription of tyrosine hydroxylase expression could not be detected in 22 blood and bone marrow specimens from normal donors, suggesting that the gene is under tight regulatory control. This finding is consistent with tyrosine hydroxylase being the rate-limiting enzyme of the catecholamine biosynthetic pathway. Thus, these findings confirm the suitability of tyrosine hydroxylase gene expression for studies involving the PCR-based detection of micrometastatic neuroblastoma in blood and bone marrow. In contrast, positive results obtained following the use of PGP9.5 as the target gene should be interpreted with caution given the frequent expression of this gene in normal cells of haematopoietic origin.

### ACKNOWLEDGEMENTS

This work was supported by grants from the National Health and Medical Research Council (Australia), the New South Wales State Cancer Council (Australia), the Leo and Jenny Leukaemia and Cancer Foundation of Australia and by the Children's Cancer Institute Australia Inc. J Gilbert is the recipient of an Australian Postgraduate Research Award.

### REFERENCES

Betz C, Papadopoulos T, Buchwald J, Dammrich J and Muller-Hermelink HK (1995) Surfactant protein gene expression in metastatic and micrometastatic pulmonary adenocarcinomas and other non-small cell lung carcinomas: Detection by reverse transcriptase–polymerase chain reaction. *Cancer Res* **55**: 4283–4286

Brodeur GM and Castleberry RP (1993) Neuroblastoma. In *Principles and Practice of Pediatric Oncology*, Pizzo PA and Poplack DG (eds), pp. 739–767. Lippincott: Philadelphia

Burchill SA, Bradbury FM, Smith B, Lewis IJ and Selby P (1994) Neuroblastoma cell detection by reverse transcriptase–polymerase chain reaction (RT–PCR) for tyrosine hydroxylase mRNA. *Int J Cancer* **57**: 671–675

Burchill SA, Bradbury FM, Selby P and Lewis IJ (1995) Early clinical evaluation of neuroblastoma cell detection by reverse transcriptase–polymerase chain reaction (RT–PCR) for tyrosine hydroxylase mRNA. *Eur J Cancer* **31A**: 553–556

Butturini A, Chen RL, Tang SQ, Hong CM, Peters J, Mathay KK, Reynolds CP and Seeger RC (1996) *Proc Am Soc Clin Oncol* **15**: 468

Chelly J, Concordet J-P, Kaplan J-C and Kahn A (1989) Illegitimate transcription: transcription of any gene in any cell type. *Proc Natl Acad Sci USA* **86**: 2617–2621

Chomczynski P and Sacchi N (1987) Single-step method of RNA isolation by acid guanidinium thiocyanate–phenol–chloroform extraction. *Anal Biochem* **162**: 156–159

Datta YH, Adams PT, Drobyski WR, Ethier SP, Terry VH and Roth MS (1994) Sensitive detection of occult breast cancer by the reverse transcriptase–polymerase chain reaction. *J Clin Oncol* **12**: 475–482

Foss AJE, Guille MJ, Occleston NL, Hykin PG, Hungerford JL and Lightman S (1995) The detection of melanoma cells in peripheral blood by reverse transcription–polymerase chain reaction. *Br J Cancer* **72**: 155–159

Ghossein RA, Scher HI, Gerald WL, Kelly WK, Curley T, Amsterdam A, Zhang Z-F and Rosai J (1995) Detection of circulating tumor cells in patients with localized and metastatic prostatic carcinoma: clinical implications. *J Clin Oncol* **13**: 1195–1200

Hoon DSB, Doi F, Giuliano AE, Schmid P and Conrad AJ (1995) The detection of breast carcinoma micrometastases in axillary lymph nodes by means of reverse transcriptase–polymerase chain reaction. *Cancer* **76**: 533–534

Krismann M, Todt B, Schroder J, Gareis D, Muller K-M, Seeber S and Schutte J (1995) Low specificity of cytokeratin 19 reverse transcriptase–polymerase chain reaction analyses for detection of hematogenous lung cancer dissemination. *J Clin Oncol* **13**: 2769–2775

Mattano LA, Moss TJ and Emerson SG (1992) Sensitive detection of rare circulating neuroblastoma cells by the reverse transcriptase–polymerase chain reaction. *Cancer Res* **52**: 4701–4705

Miyajima Y, Kato K, Numata S-I, Kudo K and Horibe K (1995) Detection of neuroblastoma cells in bone marrow and peripheral blood at diagnosis by the reverse transcriptase–polymerase chain reaction for tyrosine hydroxylase mRNA. *Cancer* **75**: 2757–2761

Mori M, Mimori K, Inoue H, Barnard G.F, Tsuji K, Nanbara S, Ueo H and Akiyoshi T (1995) Detection of cancer micrometastases in lymph nodes by reverse transcriptase–polymerase chain reaction. *Cancer Res* **55**: 3417–3420

Moss TJ and Sanders DG (1990) Detection of neuroblastoma cells in blood. *J Clin Oncol* **8**: 736–740

Naito H, Kuzumaki N, Uchino J, Kobayashi R, Shikano T, Ishikawa Y and Matsumoto S (1991) Detection of tyrosine hydroxylase mRNA and minimal neuroblastoma cells by the reverse transcription–polymerase chain reaction. *Eur J Cancer* **27**: 762–765

Noguchi S, Aihara T, Nakamori S, Motomura K, Inaji H, Imaoka S and Koyama H (1994) The detection of breast carcinoma micrometastases in axillary lymph nodes by means of reverse transcriptase–polymerase chain reaction. *Cancer* **74**: 1595–1600

Noonan K, Beck C, Holtzmayer T, Chin JE, Wunder JS, Andrulis IL, Gazdar AF, Willman CL, Griffith B, Von Hoff DD and Roninson IB (1990) Quantitative analysis of MDR1 (multidrug resistance) gene expression in human tumors by polymerase chain reaction. *Proc Natl Acad Sci USA* **87**: 7160–7164

Sarkar G and Sommer SS (1989) Access to a messenger RNA sequence or its protein product is not limited by tissue or species specificity. *Science* **244**: 331–334

Smith MR, Biggar S and Hussain M (1995) Prostate-specific antigen messenger RNA is expressed in non-prostate cells: implications for detection of micrometastases. *Cancer Res* **55**: 2640–2644

Thompson RJ, Doran JF, Jackson P, Dhillon AP and Rode J (1983) PGP9.5 – a new marker for vertebrate neurons and neuroendocrine cells. *Brain Res* **278**: 224–228

Wilson POG, Barber PC, Hamid QA, Power BF, Dhillon AP, Rode J, Day INM, Thompson RJ and Polak JM (1988) The immunolocalization of protein gene product 9.5 using rabbit polyclonal and mouse monoclonal antibodies. *Br J Exp Pathol* **69**: 91–104

Wood DP, Banks ER, Humphries SL, Kryscio RJ and Rangnekar VM (1993) Comparison of immunohistochemistry and polymerase chain reaction amplification of mRNA in the detection of prostate cancer micrometastases. *Proc Am Assoc Cancer Res* **34**: 190

© Cancer Research Campaign 1997